# Population structure and genetic connectivity of *Plasmodium falciparum* in pre-elimination settings of Southern Africa

**DOI:** 10.3389/fepid.2023.1227071

**Published:** 2023-08-01

**Authors:** Hazel B. Gwarinda, Sofonias K. Tessema, Jaishree Raman, Bryan Greenhouse, Lyn-Marié Birkholtz

**Affiliations:** ^1^ Malaria Parasite Molecular Laboratory, Department of Biochemistry, Genetics and Microbiology, Institute for Sustainable Malaria Control, University of Pretoria, Pretoria, South Africa; ^2^ EppiCenter, Division of HIV, Infectious Diseases, and Global Medicine, Department of Medicine, University of California San Francisco, San Francisco, CA, United States; ^3^ Laboratory for Antimalarial Resistance Monitoring and Malaria Operational Research (ARMMOR), Centre for Emerging Zoonotic and Parasitic Diseases, A Division of the National Health Laboratory Service, National Institute for Communicable Diseases, Johannesburg, South Africa; ^4^ Faculty of Health Sciences, Wits Research Institute for Malaria, University of Witwatersrand, Johannesburg, South Africa

**Keywords:** *Plasmodium falciparum*, genetic diversity, genetic connectivity, cross-border, Southern Africa, interconnectivity, malaria elimination

## Abstract

To accelerate malaria elimination in the Southern African region by 2030, it is essential to prevent cross-border malaria transmission. However, countries within the region are highly interconnected due to human migration that aids in the movement of the parasite across geographical borders. It is therefore important to better understand *Plasmodium falciparum* transmission dynamics in the region, and identify major parasite source and sink populations, as well as cross-border blocks of high parasite connectivity. We performed a meta-analysis using collated parasite allelic data generated by microsatellite genotyping of malaria parasites from Namibia, Eswatini, South Africa, and Mozambique (*N* = 5,314). The overall number of unique alleles was significantly higher (*P* ≤ 0.01) in Namibia (mean *A* = 17.3 ± 1.46) compared to South Africa (mean *A* = 12.2 ± 1.22) and Eswatini (mean *A* = 13.3 ± 1.27, *P* ≤ 0.05), whilst the level of heterozygosity was not significantly different between countries. The proportion of polyclonal infections was highest for Namibia (77%), and lowest for Mozambique (64%). A was significant population structure was detected between parasites from the four countries, and patterns of gene flow showed that Mozambique was the major source area and Eswatini the major sink area of parasites between the countries. This study showed strong signals of parasite population structure and genetic connectivity between malaria parasite populations across national borders. This calls for strengthening the harmonization of malaria control and elimination efforts between countries in the southern African region. This data also proves its potential utility as an additional surveillance tool for malaria surveillance on both a national and regional level for the identification of imported cases and/or outbreaks, as well as monitoring for the potential spread of anti-malarial drug resistance as countries work towards malaria elimination.

## 1. Introduction

Progress towards malaria elimination (i.e., halting malaria transmission within a country's border) around the world has been increasing steadily, with 23 countries achieving 3 consecutive years of zero indigenous malaria cases between 2000 and 2020, and 12 of these countries being certified malaria-free by the World Health Organization (WHO) during the same period (1). Since 2010, the number of malaria cases in the 21 countries selected as part of the eliminating countries for 2020 (E-2020) also decreased by 8.4% (1). Based on this progress, 8 additional countries joined the elimination initiative in 2021, bringing the current total to 25 countries that are now part of a renewed initiative to eliminate malaria from within their borders by 2025 (E-2025) (2). Movement in reducing the number of malaria cases and deaths globally has however generally stalled in recent years, particularly in Africa where the majority of these cases and deaths originate.

The southern Africa region accounts for roughly 10% of the 228 million cases reported in the WHO African region with the majority of these cases (79%), coming from 2 high transmission countries—Mozambique and Angola—primarily caused by *Plasmodium falciparum* (1, 3). It is because of this relatively low case load in southern Africa compared to the rest of the continent that the region was earmarked for malaria elimination by the WHO (4). Three countries—Botswana, Eswatini, and South Africa—were identified as having the potential to eliminate malaria as part of both the E-2020 and E-2025 initiatives (1). Countries within southern Africa, however, came to the early realization that malaria elimination in any one southern African country would be challenging without regional cooperation and collaboration (5, 6) due to the high levels of interconnectedness between the countries (7, 8). This interconnectedness is caused mainly by highly mobile migrant human populations who facilitate the constant movement of malaria parasites across country borders, mostly from high-transmission countries to low-transmission countries in the region (7, 8).

**Figure 1:**
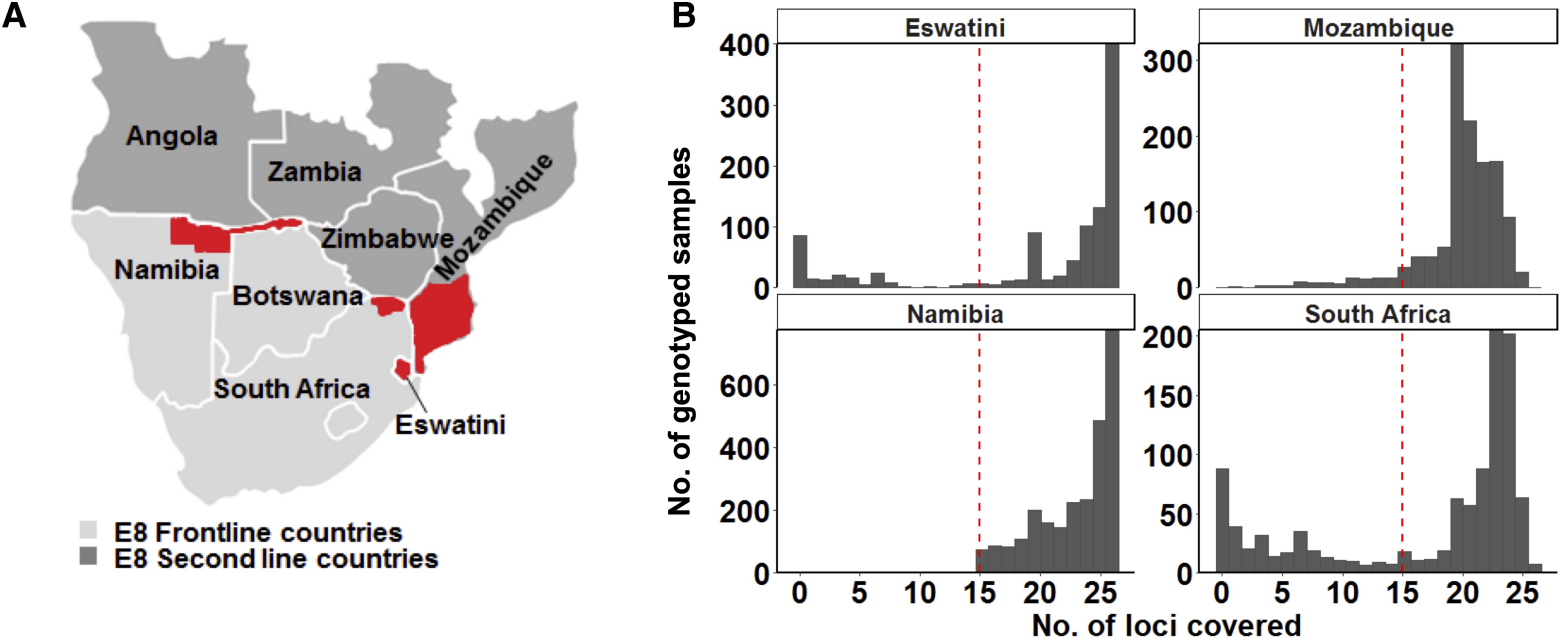
Study area and design. (**A**) Map of the study site showing the countries from where genotyping data was collected. The specific areas where samples were collected are coloured in red on the map: the north-eastern border region of Namibia (the Kavango and Zambezi regions), the north-eastern border region of South Africa (the Vhembe District in the Limpopo Province), the whole of Eswatini and parts of southern Mozambique. Study sites represent countries in the Southern Africa Elimination-8 region. (**B**) Only samples that met the 60% genotyping coverage threshold were analysed. The red dashed line indicates the threshold which represents alleles being detected on at least 15 of the 26 loci.

Therefore, as part of collaborative regional malaria elimination efforts, the Elimination 8 Initiative (E8) was initiated in southern Africa to lead malaria elimination on the continent (9). This includes four low-transmission front-line countries (South Africa, Namibia, Eswatini, and Botswana) that are envisaged to pave the way for another four higher transmission, second-line countries (Mozambique, Zimbabwe, Zambia, and Angola) (4, 9). To achieve this, one of the core objectives of the E8 is preventing cross-border malaria transmission (9), however, this is in context of the unique challenge that the front-line countries share porous borders with areas of higher transmission (Figure 1A), associated with both human migration and mosquito movement (7, 10). Although the implementation of five cross-border malaria control initiatives and the deployment of malaria health units at strategic points along shared borders in the southern African region led to a 30%–46% reduction in malaria incidence and mortality (9), front-line countries in the region have not been able to reach their elimination goals at the initially proposed target dates (11, 12).

The potential impact of imported infections on local transmission is an important consideration for eliminating countries that share porous borders with areas of higher transmission, as importation can play a significant role in sustaining or re-establishing local transmission (9). Strategic priorities therefore include understanding regional connectivity in infections, identifying source and sink parasite populations in the region, and cross-border blocks of high parasite connectivity to inform intervention harmonization and synchronization. Identifying blocks of high parasite connectivity within and across a country's borders and coordinating elimination strategies accordingly would therefore accelerate success towards elimination on both a national and regional level.

Parasite population genetics may help improve surveillance efforts and understand regional transmission dynamics. Lessons have been learnt from other diseases such as the polio eradication programme, outbreaks of Ebola, and ongoing transmission of tuberculosis by connecting genetic epidemiology and disease surveillance (13). Most recently, the coronavirus disease 2019 (COVID-19) pandemic showed how genomics can be used to identify sources of disease outbreaks, track, and trace infections, as well as identifying imported infections in supporting the timely control of the disease (14–20). In the malaria field, countries such as China recently achieved elimination status with genomic surveillance at the core of their elimination programme (21). This can also be achieved in southern Africa.

Previous studies on malaria parasite population genetics have portrayed the parasite population in Africa as a single contiguous population (22–25) until recently (26) when signatures of parasite population structure were observed/reported, suggesting that the parasite population is in fact fragmented. This fragmentation, which clustered *P. falciparum* parasite populations in sub-Saharan Africa into major western, central, and eastern regional subgroups as well as a highly divergent Ethiopian subpopulation, has been attributed to the parasite's ancestry associated to the respective regional blocks which corresponded with both the parasite's origin and with historical human population movement and the use of interventions that may also drive the selection of for example drug-resistant parasite strains (26). The southern African parasite population was, however, inadequately represented in sub-Saharan African parasite population studies with only Zimbabwe and Malawi (22, 26) from the southern African region included.

Limited *P. falciparum* population genetics studies that have been performed in low-transmission settings of southern Africa include Namibia where moderate to high parasite genetic diversity, fine-scale parasite population structure, and cross-border parasite genetic connectivity with neighboring higher transmission countries (Zambia and Angola) (7) was identified. Similarly, Zambia identified cross-border parasite genetic connectivity with neighboring higher transmission country of the Democratic Republic of Congo (27), and Eswatini with neighboring Mozambique linked through travel history data (10). Some of the studies showed that the level of parasite genetic diversity does not necessarily reflect the transmission intensity in the country possibly due to the importation of malaria from higher transmission countries (10). This contrasted with high transmission countries studied extensively in sub-Saharan Africa where the high transmission intensity has been reflected by high levels of parasite genetic diversity (22–24, 28, 29). If measures of parasite genetic diversity are to have utility in assessing the level of transmission intensity in low transmission settings in southern Africa, then parasites in this region should be assessed together to identify the best parasite genetic diversity metrics to be used as indicators of transmission intensity in the region. Even more important is to define the extent to which these metrics can be used at all, in which settings, and with which caveats, as they may not be informative in all settings, at least without proper context. This study, therefore, presents an initial attempt to identify the parasite genetic diversity metrics that would be the best indicators of transmission intensity within the southern African region.

Thus, this study set out to perform a meta-analysis by comparing parasite populations recently genotyped from South Africa (30, 31), to those from neighboring countries (Namibia, Eswatini, and Mozambique) (7, 10) where similar technology was used, to understand how the South African parasite population compares to that of other parasite populations in the southern African region as the country and the region work towards malaria elimination. This study aimed to evaluate the population structure, genetic connectivity, and gene flow patterns between different *P. falciparum* populations from the selected countries in the southern African region. Additionally, parasite genetic metrics that would be the most useful to assess transmission intensity based on the collective genetic diversity represented by parasites in the southern African region were evaluated.

## 2. Methods

### 2.1. Ethical approval

Ethical approval for the study was obtained from the University of Pretoria, Faculty of Health Sciences Research Ethics Committee (Ethics Reference No. 406-2014), and the Limpopo Department of Health (Ref: LP_201906_011). The National Institute for Communicable Disease holds ethical approval for analysis of the KwaZulu-Natal (KZN) samples from the KwaZulu-Natal Provincial Department of Health, the Health Ethics Review Committee of the University of Witwatersrand (M170869), and Advarra Research Compliance Solutions (Maryland, USA). The other collated genetic data were obtained based on ethical approval and protocols of the local National/Institutional ethical review committees of Namibia (7), Eswatini (10), and Mozambique.

### 2.2. Datasets used

A meta-analysis was conducted based on the collated data from publications of parasite population genetics studies conducted in Namibia (symptomatic cases identified at outpatient clinics of 29 health facilities in two regions of northeastern Namibia with the country's highest burden: Kavango East and Zambezi, collected from February 2015 to June 2016) (7), Eswatini (all national symptomatic cases collected from July 2014 to July 2016) (10), and South Africa (symptomatic cases collected throughout each year from 2016 to 2018 at randomly selected high burden health facilities in the Limpopo Province with the country's highest burden of malaria) (30) and data generated from Mozambique [(unpublished—symptomatic cases from health facilities at the country's lowest burden southern region, collected from 2014 to 2016; published—asymptomatic cases collected at the border of South Africa's KZN province and Mozambique's southern region, over a 6-week period in February and March 2018 (31)] (Figure 1A). The genotyping data were comparable in terms of the same microsatellite genotyping technology and equipment used to generate it and similar microSPAT software settings used for allele calling for all samples collectively as described previously (7, 10, 30).

Quality control measures were implemented to assess the DNA samples, marker reliability, and technical errors during genotyping. The MicroSPAT software (available at the GitHub link: https://github.com/Greenhouse-Lab/MicroSPAT/releases/tag/v2.0.3) was used to automate the identification of true alleles and distinguish actual peaks from artifacts in the electropherograms obtained. This was achieved by employing a classifier algorithm that considered the position and size of locus-specific patterns in relation to a primary peak) (30). If minor peaks reached a height of at least one-third of the major peak, multiple alleles per locus were recorded. The genotyping data from all samples were then combined and processed using the microSPAT software with consistent settings. A semi-supervised naïve Bayes classifier was employed to ensure consistency in allele calling and mitigate variability. For inclusion in subsequent population genetics analysis, samples had to achieve a genotyping coverage of at least 60% (with alleles detected on 15 or more loci) (30).

### 2.3. *P. falciparum* population-level diversity in Southern Africa

On a population level, the heterozygosity, number of unique alleles per locus (allelic richness), and multilocus linkage disequilibrium (using clone corrected data) for each population of isolates defined by the geographical location of sample collection sites (countries) was calculated in R using the *poppr* package as described previously (30). ANOVA pairwise *t*-tests were used to compare differences in population level (He) diversity between the 4 countries/populations and assess whether parasite population-level genetic diversity reflects the transmission intensity observed in the four countries.

Since allelic richness is biased by sample size, to assess the distribution of alleles across the populations and the number of alleles private to each population with a standardized sample size, the ADZE software (32) was used. To compensate for differences in sample sizes, a rarefaction approach that considers the maximum equal-sized sub-samples from each population was considered. Genetic bottlenecks of the parasite populations and linkage disequilibrium (LD) were determined respectively and compared between the different parasite populations per country.

### 2.4. *P. falciparum* within-host diversity in Southern Africa

For the within-host diversity, the MOI and Fws index were calculated as previously described (7, 10, 30). ANOVA pairwise *t*-tests were used to compare differences in within-host (MOI and 1-F_WS_) diversity between parasite populations from the four countries and assess whether within-host diversity reflects the transmission intensity observed in the four countries.

### 2.5. Parasite population structure and differentiation within Southern Africa

To determine signatures of population structure between the four countries, Discriminant Analysis of Principle Components (DAPC) was performed using the *adegenet* package in R software (33, 34) with countries used as priori groups. A scatterplot of the first and second linear discriminants of DAPC was then plotted. To prevent the overfitting of clusters, the optimal number of principal components (PC) to be retained was confirmed by cross-validation of the DAPC. Cross-validation provides an objective optimization procedure for identifying the “goldilocks point” in the trade-off between retaining too few and too many PCs in the model. Data was divided into a training set (90% of data), and a validation set (10% of data), and members of each of the identified clusters were stratified by random sampling to ensure that at least one member of each group or population in the original data is represented in both training and validation sets. DAPC was then performed on the training set with variable numbers of PCs retained. The extent to which the analysis was able to accurately predict group memberships of individuals in the validation set was used to identify the optimal number of PCs to be retained. Sampling and DAPC procedures were repeated 30 times at each level of PC retention, and the optimal number of PCs retained was associated with the lowest root mean square error. Population differentiation between the geographic areas was also determined by measuring pairwise measuring Wright's F-statistics (F*_ST_*), using the *adegenet* package (34) in R. Hendrick's G*_ST_* and *Jost's D*, were calculated using the *mmod* package (35) in R. The Monte Carlo method was used to test the significance of pairwise FST between the countries by completing 999 permutations. An isolation-by-distance approach that correlates genetic distance to geographic distance was used to test the significance of population structure. The Monte Carlo method was used to test the significance and was based on 999 replicates.

### 2.6. Parasite genetic connectivity and gene flow within Southern Africa

To examine the genetic connectivity of parasite genotypes across countries, the number and proportional distribution of multi-locus genotypes (MLG) genotypes as well as genotypes per locus shared across populations was assessed. The extent and direction of parasite gene flow within and between countries were then determined to infer parasite migration patterns among populations and identify cross-border blocks of high parasite connectivity to identify the major source and sink areas in the region. Estimates of historical gene flow patterns were made using co-dominant diploid data and divMigrate online software (36) (https://popgen.shinyapps.io/divMigrate-online/). Asymmetric bidirectional gene flow was assumed. Relative migration and gene flow was determined based on Wright's equation:

$$\mathrm{FST}=\;\frac{1}{4}Nem+1$$

where *Ne* = population size and *m* = gene flow (37). The migration patterns between different parasite populations were tested for different levels of gene flow calculated using *Jost's D* method (38).

To estimate more recent migration or gene flow patterns across countries the BayesAss v3.0.4 program (39) was used, again with dominant allele data. Bayesian inference with Markov chain Monte Carlo (MCMC) simulations were used to estimate the fraction of immigrants per population. Mixing parameters for migration rates, allele frequencies, and inbreeding coefficients were optimized to ensure that the acceptance rates for each parameter were between the recommended target ranges of 20%–60% (39). To calculate the average of gene flow estimates, MCMC simulations were performed using 107 iterations, with a burn-in of 106 and a sampling interval of 100.

## 3. Results

### 3.1. Meta-analysis data population

To ensure that a good representation of sample size across the 4 selected countries would be achieved, all the data from the selected studies were used in this meta-analysis. A total of 5,314 samples of curated genotype data were collected from publications from Namibia (*n* = 2,585) (7), Eswatini (*n* = 835) (10), and South Africa (*n* = 747) (30) and unpublished data from Mozambique (*n* = 1,147) (Figure 1A). Additionally, 46 of the samples in the Mozambique dataset were obtained from published data (31). A genotyping coverage threshold of ≥60% where alleles in each sample had to be detected at a minimum of at least 15 loci was maintained for the downstream population genetics analysis (Figure 1B). Therefore, allelic data from as many good-quality genotyped samples as could be accessed, was extracted for each country.

### 3.2. Allelic patterns across Southern African parasite populations

**Figure 2:**
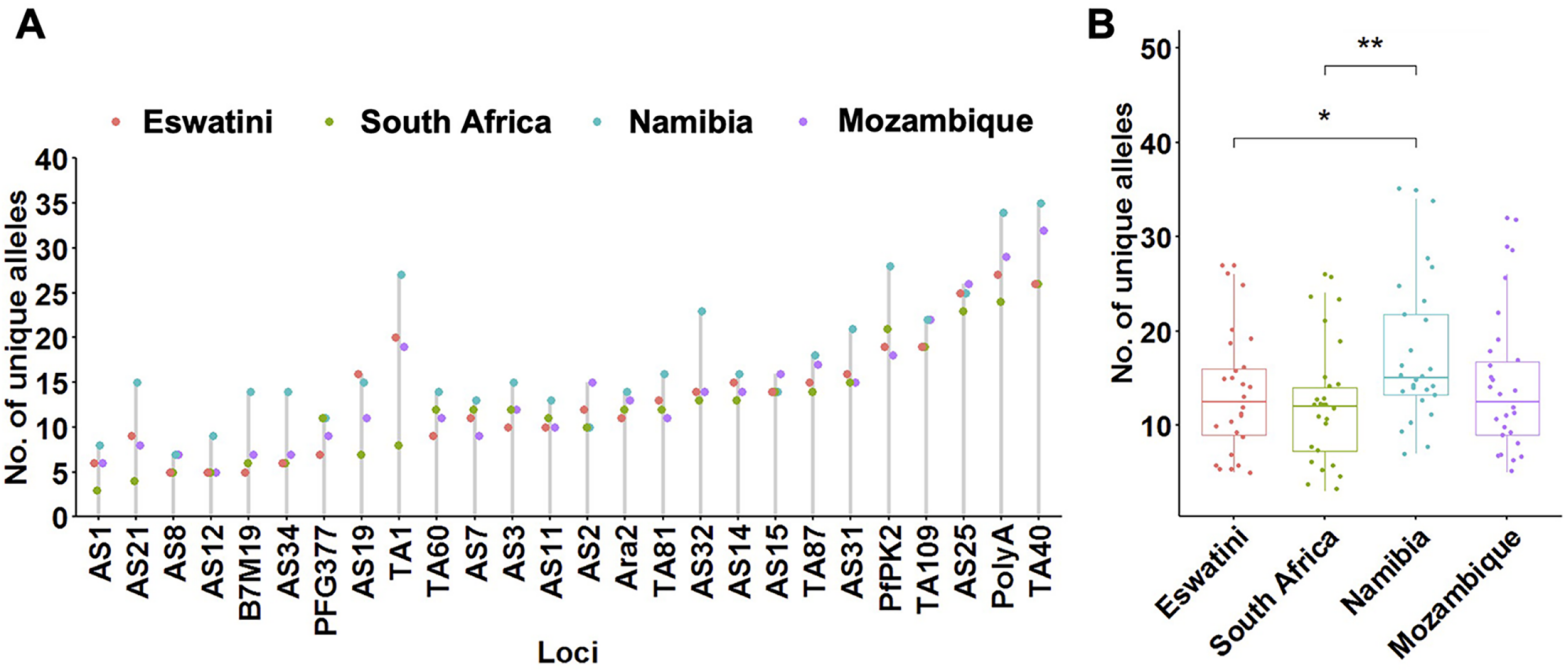
Allelic patterns across Southern African parasite populations. (**A**) Distribution of the number of unique alleles per locus across countries. (**B**) Comparison of the allelic richness between countries. The corresponding box plots show summary statistics with jitters indicating distribution of individual data points. Pairwise *p*-values (*t*-test) of the number of unique alleles compared between countries are indicated where, not significant (ns): *p *> 0.05; *p ≤ 0.05; **p ≤ 0.01.

To determine patterns of allelic richness in parasites from the 4 countries in comparison to each other, frequency distribution of the number of unique alleles across all loci and pairwise ANOVA analyses were performed. Allelic distribution across loci generally showed a similar trend in the number of unique alleles identified at each locus per country (Figure 2A). Exceptions were locus TA1 which showed a much lower number of unique alleles in South Africa compared to the other 3 countries; and loci PfPK2, AS32, AS34, and B7M19 which had a much higher number of unique alleles identified in Namibia compared to the other countries. The overall number of unique alleles was significantly higher (*P* ≤ 0.01, pairwise *t*-test) in Namibia (mean *A* = 17.3 ± 1.46) compared to South Africa (mean *A* = 12.2 ± 1.22) and Eswatini (mean *A* = 13.3 ± 1.27) (*P* ≤ 0.05, pairwise *t*-test), and was not significantly (*P* > 0.05, ANOVA pairwise *t*-test) different between the other country pairs including Mozambique which had a mean *A* = 14.0 ± 1.38 (Figure 2B). This was based on both uncorrected and adjusted/standardized sample sizes. Allelic richness patterns, therefore, did not reflect transmission intensity in the region.

### 3.3. Parasite population level diversity is high and stable and does not reflect transmission intensity in Southern African

To determine which metrics of parasite population level diversity were good indicators to reflect transmission intensity in the region, heterozygosity and the numbers of unique haplotypes were assessed within and between countries in the region. Evidence of parasite population bottlenecks was also assessed. The level of heterozygosity was high (mean He = 0.75) but did not differ significantly between all countries (*P* > 0.05, ANOVA pairwise *t*-test) (Table 1). Therefore, this may suggest that the level of heterozygosity does not reflect transmission intensity and may not necessarily be a good indicator of transmission intensity in the southern African regional parasite populations. Eswatini had the highest proportion of identical haplotypes (2.2%, 18/835) in its population, followed by South Africa (0.9%, 7/747), Mozambique (0.7%, 8/1,147) and Namibia (0.3%, 9/2,585) (Table 1). Significant linkage disequilibrium (LD) (*P* ≤ 0.001, Monte Carlo test, 1,000 permutations) was detected in all four countries, which suggest some level of local transmission (Table 1). Multilocus LD was also detected in the overall southern African population (LD = 0.18; *P* ≤ 0.001, Monte Carlo test), which may suggest some level of population structure. Surprisingly, the highest LD was observed in Namibia (LD = 0.212) although it had the least number of identical haplotypes. This inferred relatedness may indicate genetically related clusters due to rapid reduction or expansion in transmission and effective parasite population size influenced by different processes.

**Table 1:** Genetic diversity of *Plasmodium falciparum* parasite populations from pre-elimination settings in Southern Africa.

Population	*n*	*h*	A ± SE	He ± SE	MOI ± SE	1-Fws ± SE	LD
Eswatini	835	817	13.3 ± 1.3	0.75 ± 0.03	2.32 ± 0.04	0.22 ± 0.01	0.17***
South Africa	747	740	12.2 ± 1.2	0.74 ± 0.03	2.13 ± 0.04	0.21 ± 0.01	0.14***
Namibia	2,585	2,576	17.3 ± 1.45	0.75 ± 0.03	2.74 ± 0.03	0.22 ± 0.01	0.212***
Mozambique	1,147	1,139	14.0 ± 1.38	0.74 ± 0.03	2.14 ± 0.03	0.24 ± 0.01	0.119***
Total	**5,314**	5,272	**14.2 **±** 1.3**	**0.75 **± **0.03**	**2.33 **± **0.03**	**0.22 **±** 0.01**	**0**.18***

*n*, number of isolates genotyped; *h*, number of haplotypes (or multilocus genotypes); A, mean number of alleles per locus; He, heterozygosity; MOI, multiplicity of infection; 1-Fws, outbreeding, LD, linkage diseqilibrium.

*** *p* ≤ 0.001 (Monte Carlo test, 1,000 permutations).

Allele frequency distribution showed L-shaped mode shift graphs for parasites from all four countries, which suggests that there was no evidence for recent parasite genetic bottlenecks in any of the parasite populations as expected under the assumption of mutation drift equilibrium. This lack of bottlenecks can be attributed to the importation of malaria parasites, especially from high-transmission countries. Most alleles were found in the rarest class with allele frequencies ≤0.10 as is expected from neutral evolution. This, therefore, suggests that the higher proportion of shared alleles observed in the low-transmission countries (Eswatini, South Africa, and Namibia) as observed by LD may not have been due to recent intervention but rather had been sustained from previous years before sampling was done. Estimates of effective population size (*Ne*) were highest in Namibia (3,148), followed by Mozambique (1,206), Eswatini (1,027), and South Africa (847) which correlated to the different sample sizes. This indicates high parasite population diversity. Overall, this data shows that the genetic diversity of the parasite population in these countries is high and stable. Therefore, these metrics of parasite population genetics may not be the best at reflecting transmission intensity in the region.

### 3.4. Parasite within-host diversity may highlight co-transmission or superinfection in Southern Africa

**Figure 3:**
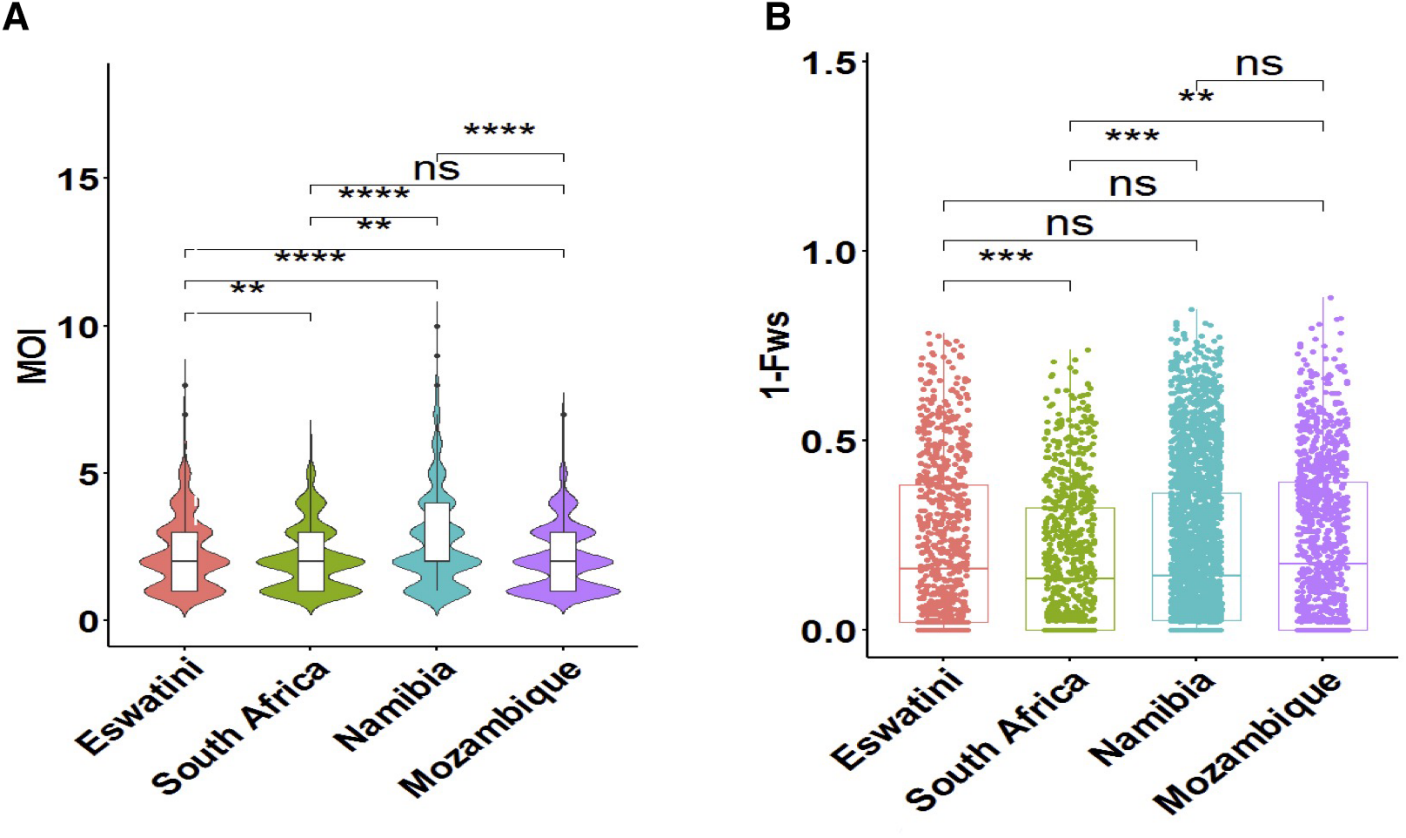
Within-host diversity described in relation to transmission intensity across the different endemic countries. (**A**) The violin plots showing full distribution of the data at different MOI values are colour coded to correspond to the associated country. The corresponding box plots show summary statistics (i.e., the median and box indicating the 25th and 75th percentile interquartile ranges; with dots representing potential outliers). (**B**) Within-host diversity index (1-Fws) describing outbreeding. The corresponding box plots show summary statistics with jitters indicating distribution of individual data points. Pairwise *p*-values (*t*-test) of MOI and 1-Fws compared between countries are indicated where, not significant (ns): *p* > 0.05; *p ≤ 0.05; **P ≤ 0.01; ***p ≤ 0.001; ****p ≤ 0.0001.

To determine whether metrics of within-host diversity were better indicators to reflect transmission intensity in the region, MOI distribution, and level of outbreeding were evaluated within and between countries. The overall southern African population showed that the MOI in the region is moderate with a mean MOI of 2.33. MOI was significantly higher in Namibia (MOI maximum = 10) than in all other countries, confirming that infections were the most complex in the specific areas sampled in this country (Figure 3A). Interestingly, this MOI was higher than that of Mozambique (MOI maximum = 7), which is a country with higher transmission. The proportion of polyclonal infections with MOI >1 was highest at 77% for Namibia, 70% for Eswatini, 66% for South Africa, and least at 64% for Mozambique.

Although the mean MOI between parasites from South Africa and Mozambique was not significantly different (*P* > 0.05, pairwise *t*-test), the level of outbreeding was significantly higher (*P* ≤ 0.01, pairwise *t*-test) in Mozambique (Figure 3B) compared to South Africa, which may suggest different biological scenarios of the parasite in the two neighboring countries (i.e., co-transmission which may result in more related coinfecting clones *vs*. superinfection where the different clones in an individual infection may not be related). When taken up by a mosquito during a blood meal, these unrelated clones are more likely to out-cross thus generating more diverse parasites in the population. Parasites from South Africa had the highest proportion (40%, 297/747) of clonal parasites as described by 1-Fws (1-Fws < 0.05), which suggests higher levels of inbreeding in this country compared to parasites from the other three countries. This was also reflected in the smallest proportion of highly diverse (1-Fws > 0.30) parasites in South Africa (28%, 207/747) compared to Eswatini (34%, 281/835), Namibia (32%, 818/2,585) and Mozambique (37%, 425/1,147). Therefore, both the level of outbreeding and MOI highlight other underlying factors happening to the parasites in the region.

### 3.5. Significant *P. falciparum* population structure in southern Africa

To establish whether parasite populations from the different countries in the southern African region were genetically similar based on their geographic origins, population structure analysis, and genetic differentiation of parasites were performed. Genetic differentiation between pairs of parasite populations was very low as described by Nei's G*_ST_*, and ranged from −0.00008 to 0.002. *Jost's D* ranged from −0.00003 to 0.00113 which suggests that up to 0.1% of alleles were unique between the most genetically distant parasite populations (i.e., Namibia and South Africa). The distribution of private/unique alleles across the four countries showed that Namibia contained the majority of (161/214) private alleles, followed by South Africa (37/214), Eswatini (14/214), and Mozambique (2/214). Similarly, the rarefaction approach which accounted for differences in sample size confirmed that Namibia has both the highest allelic richness and the highest number of private alleles compared to the other countries. The smallest values in both categories of the mean number of distinct alleles per locus and the mean number of private alleles per locus occur in South Africa and Mozambique respectively. This private allelic richness, therefore, suggests that there is an endogenous circulation of parasites in the sampled areas of each of the countries or possibly random chance due to sampling (since overall frequencies are quite similar) or potential technical differences. Genetic distance (*Jost's D*) between parasites from the different countries was significantly positively correlated (*r*^2^ = 0.72, *P* = 0.001, Mantel test of matrix correlation) to geographic distance thus supporting that parasites were isolated based on the geographic distance between them. It is important to note that these values are all very small indicating little if any meaningful difference based on this metric.

**Figure 4:**
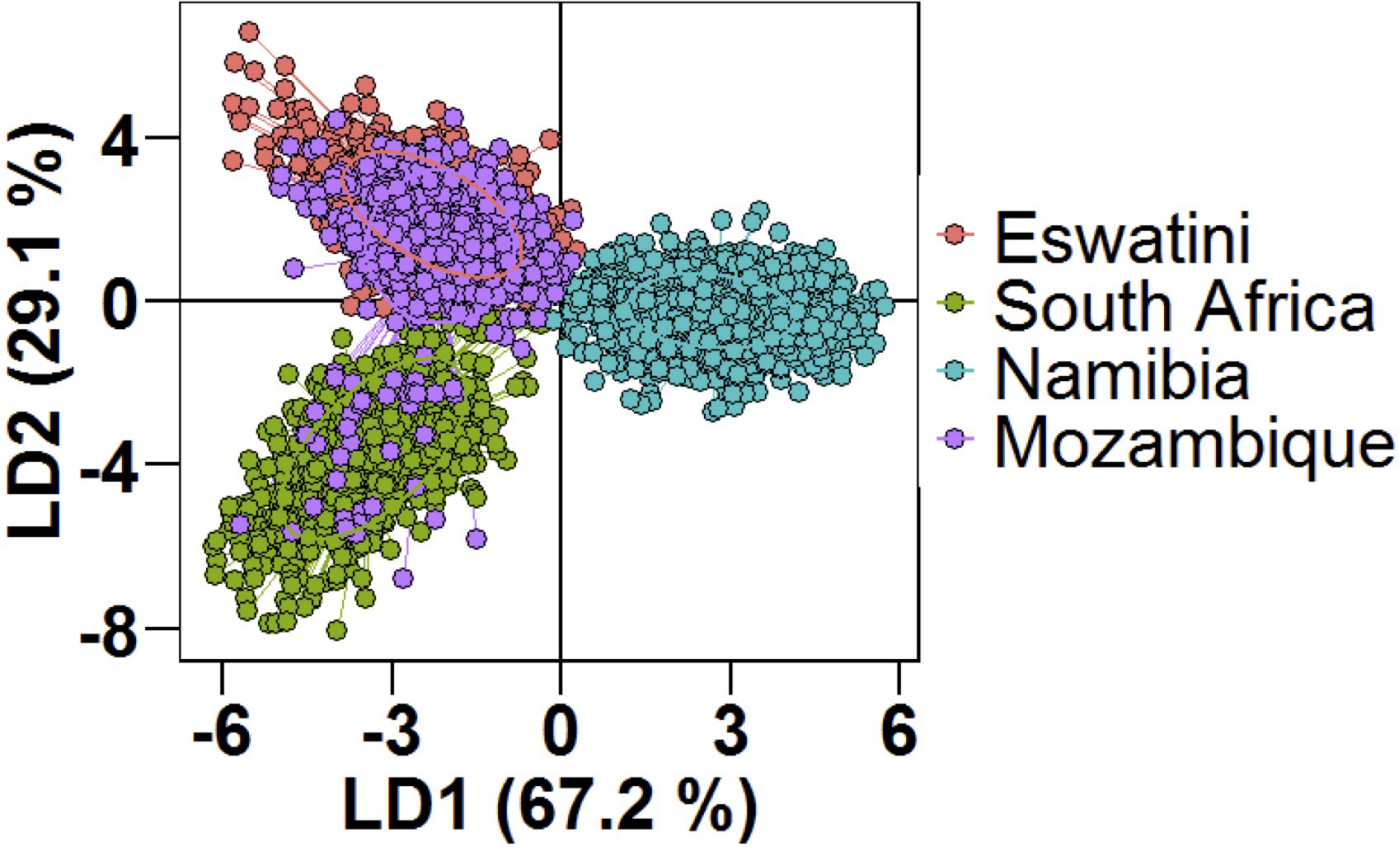
Population structure of *Plasmodium falciparum* populations in Southern Africa as described by DAPC. Scatterplot of the discriminant analysis of principal components (DAPC) based on discrimination of *P. falciparum* populations stratified by country of origin of infections. Individual multi-locus genotypes appear as dots. Colours and lines represent population membership. Analysis is based on retention of 150 principal components.

Using DAPC analysis, clear separation of the Namibian parasite population by LD1 from those in the MOSASWA (Mozambique, South Africa, and Eswatini) region was observed in the DAPC analysis (67% genetic variance explained by DA eigenvalues Figure 4), which suggests that the parasites in Namibia are genetically distinct from those in the MOSASWA block. LD2 (29.1% genetic variance explained by DA eigenvalues) separated the South African parasite population from the Mozambique and Eswatini parasite populations. Where haplotypes from the different countries overlapped, this suggests transmission connectivity supported by geographic proximity. The lack of population differentiation between Eswatini and Mozambique suggests strong gene flow between both populations. While some haplotypes from Mozambique and South Africa also overlap, this is to a lesser extent than those from Eswatini and Mozambique, which suggests a less strong gene flow between Mozambique and South Africa.

### 3.6. Evidence of cross-border parasite genetic connectivity in Southern Africa

To determine the level of the interconnectivity of parasites in the region, genetic relatedness, and connectivity between parasites from the different countries were assessed. Out of the 5,314 samples studied, there were no identical multi-locus genotypes shared across the different parasite populations from the different countries. This was expected given the high level of parasite genetic diversity in the region described previously and the high variability in microsatellite markers.

**Figure 5:**
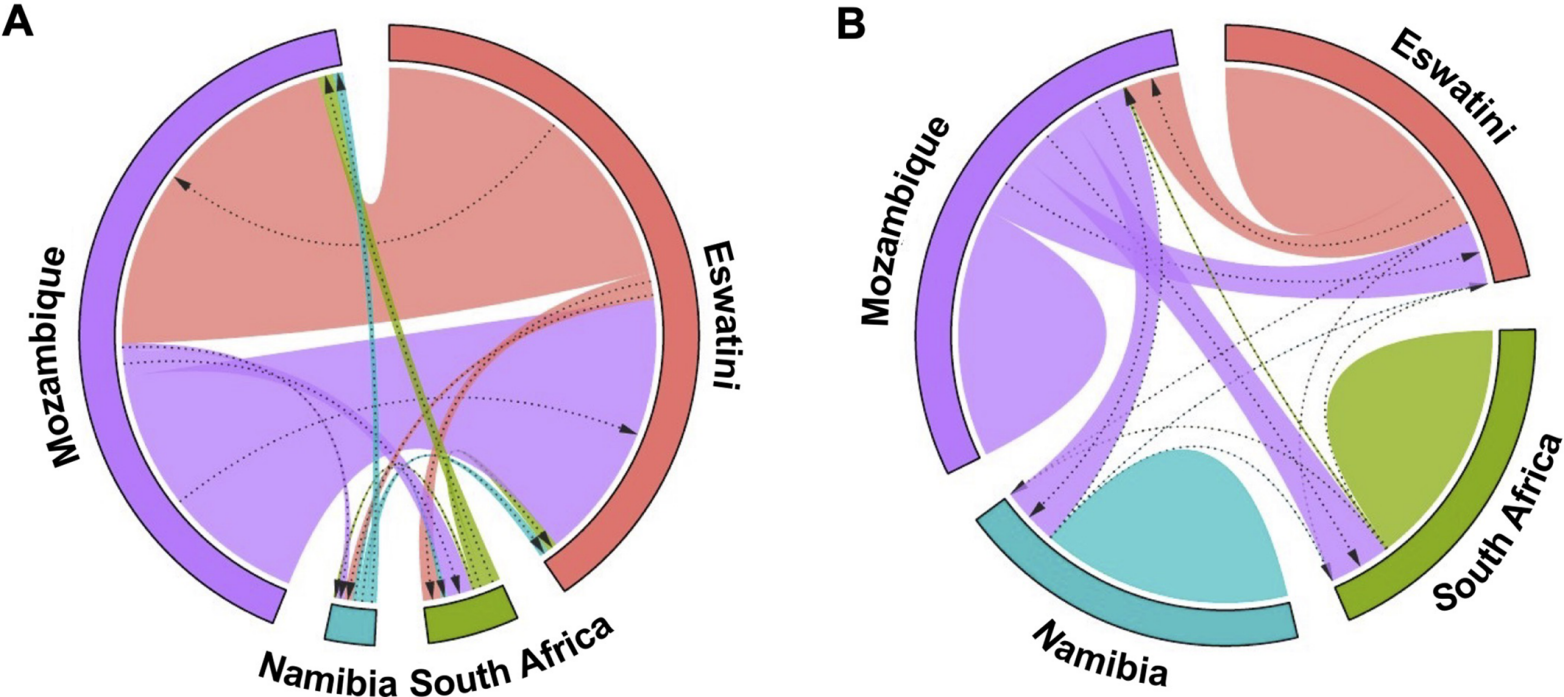
Gene flow diagrams of historic and recent inferred genetic connectivity between *P. falciparum* parasites among four countries in the Southern African Elimination-8 region. (**A**) Historical gene flow estimates were derived from divMigrate online. The relative migration values were derived from *Jost's D*. (**B**) Recent gene flow estimates were calculated using BayesAss. In both plots, arrows indicate direction of gene flow from one country to another. Arrow thickness reflects the strength of gene flow between different parasite populations. Patterns for each diagram are independent. Similar widths of arrows do not represent the same amount of gene flow across each of the two diagrams.

These results were supported by evidence of gene flow between parasites from the different countries. The greatest historical gene flow was observed between Mozambique and Eswatini (Figure 5A). Mozambique received 77% of migrants from Eswatini and Eswatini received 100% of its migrants from Mozambique as indicated by the relative migration of the parasites between the population pair determined by divMigrate online (Figure 5A). While in practice, it is very unlikely that Mozambique received many if any migrants from Eswatini given the differences in transmission intensity, it is interesting that this analysis indicates movement in this direction. The least relative migration was observed from Namibia to Eswatini (2%), Namibia to Mozambique (2%), and South Africa to Namibia (2%). Although South Africa is relatively geographically close to Eswatini and Mozambique, parasite migration to and from these countries (Eswatini and Mozambique) was relatively low and ranged between 3% and 7%. The migration patterns displayed in this plot support the population structure observations made in the DAPC analysis in Figure 4.

Patterns of recent parasite gene flow estimates based on BayesAss migration data confirmed that most of the gene flow is between Eswatini and Mozambique. Mozambique had the majority of (parasite) emigrants (21% to Eswatini, 20% to South Africa, and 19% to Namibia) (Figure 5B) compared to any other country which supports that it is the major source country. Eswatini on the other hand received most of its migrants from Mozambique (18%) compared to 0.1% from South Africa and 0.02% from Namibia. Namibia and South Africa both received the least migrants of less than 1% from each of all the other countries. The “humps” in Figure 5B represent gene flow originating from within the same country which is indicated in all 4 countries. This supports the endogenous circulation of parasites revealed by private allelic data in each of the countries. Overall, these analyses showed gene flow between *P. falciparum* parasite populations in all four countries which imply interconnectivity between the parasite populations. Additionally, there appeared to have been considerably more gene flow between the countries historically than recently which may have been influenced by the implementation of control interventions and cross-border interventions.

## 4. Discussion

The potential use of *P. falciparum* population genetics tools in elimination efforts in southern Africa is now gaining momentum. Findings from this study demonstrate strong evidence for parasite population structure in the southern African region and provide insight into patterns of parasite gene flow and connectivity within and across national borders of the selected endemic countries in the E8 region. This data demonstrates the potential of efficiently generating genetic information with similar shared criteria that can be used for regional malaria control and elimination efforts, as well as the potential utility of supplementing traditional surveillance data with molecular surveillance data generated by direct evaluation of the parasite population genetics.

The performance of both indices of parasite within-host and population-level diversity in measuring the level of transmission intensity in the southern African parasite populations as shown in this study highlights the complexity of transmission dynamics in such highly interconnected areas in the region. This study has shown that the maintenance of high levels of parasite genetic diversity may be a critical barrier to malaria elimination in this region. Connectivity between endemic and pre-elimination areas in the region aided by human migration of infected individuals may offset the effect of geographic fragmentation in parasite populations thus enabling re-introduction to areas where the disease may already have been controlled. Given the limited sampling parameters outlined in this study, metrics of transmission intensity were often generalized to represent the entire country. However, in order to make valid comparisons, it is necessary to make use of local metrics of transmission intensity that take into account both the temporal and spatial factors. Comparing genetic data from independent studies from different countries can provide validation and replication of findings. Replicating analyses using different datasets enhances the robustness of the results and helps identify consistent patterns or novel insights that cut across specific study settings.

The greatest allelic richness was observed in Namibia, which was unexpected since Mozambique is the highest transmission country of the 4 study countries. However, this observation may be explained by the fact that the southern region of Mozambique where most study samples may have been collected from has a much lower transmission intensity for example in Maputo city than other areas in Mozambique where transmission intensity is much higher such as Cabo Delgado Province (40). Additionally, some of the highest transmission areas in Namibia were sampled during an “outbreak” year. Transmission intensity is, however, still much higher in Mozambique than in Namibia, and the observed differences could have been due to other aspects such as sample size. Another alternative is that maybe allelic diversity is not only affected by transmission intensity. Population movement in the study area as well as settlement factors such as how clustered homesteads and villages play a role. The closer they are, with lower population movement, can lead to circulation of a few clones. Another consideration is that samples from Namibia show greater diversity due to importation from Angola rather than because of transmission intensity.

Genetic data confirmed the high level of gene flow particularly in the MOSASWA block, which is possibly linked to the geographic proximity of the countries and human migration for economic reasons (8). Relative migration analysis of multilocus haplotypes allowed investigation of the strength and direction of parasite flow amongst the parasite populations from the four different countries. This showed more parasite migration between Mozambique and Eswatini, and relatively limited migration to and from South Africa and Namibia which was consistent with genetic differentiation, private allelic, and clustering analyses. Similar trends of the lack of separation between parasites from countries with different malaria transmission intensities sharing borders in sub-Saharan Africa were also observed between Zambia (moderate transmission) and the Democratic Republic of the Congo (high transmission) (27). This was also true for Namibia (low transmission) and higher-transmission neighbors Angola and Zambia (7). There was no evidence of parasite population structure observed between the countries involved suggesting contiguous transmission zones in those areas (7, 27). Separation of the Namibia parasites from those in the MOSASWA block suggests interrupted clades of gene flow and provides evidence that some lineages/haplotypes may be unique to the different geographical locations (countries). Another possible explanation for this observation is that the MOSASWA block is a contiguous area whilst Namibia is more distant. This evidence was supported by the shared parasite genotypes at most of the loci thus inferring genetic connectivity between geographically distinct locations.

The wide dispersal of shared parasite genotypes across the different countries suggests that connectivity among the different endemic areas, likely caused by human migration (8) (since mosquitoes cannot fly over such long distances), sustains disease transmission in the region hindering elimination efforts. Additionally, the close geographic proximity of the South African hotspot area where the majority of samples were collected (30), to the Zimbabwean southern border region and the direct transport route that links the two countries raises a question of whether parasites from Zimbabwe instead may be more similar to those in the Limpopo Province of South Africa and may be the greater source of imported infections seeding local transmission in that area. Alternatively, it may also be possible that there is not a lot of importation in this area and that there is primarily sustained local transmission (30). Unfortunately, there were no samples genotyped using the same criteria as that of all other samples in this study that represented the Zimbabwean or other neighboring country parasite populations at the time this analysis was done to enable us to make this assessment. However, in low transmission settings, importation from higher transmission settings leads to seeding events which then become local transmission.

Overall, these analyses showed gene flow between *P. falciparum* parasite populations in all four countries which imply interconnectivity between the parasite populations. The results, based on the analyzed sample set suggest that Mozambique and Eswatini parasite populations are the most genetically connected. This confirms the importation reported between these two countries based on patient travel history information. The limited gene flow to and from South Africa (Vhembe District, Limpopo Province) and Namibia also confirms the high level of local transmission as reported through patient travel data (7, 30). Although the signals of gene flow to and from these areas were not very strong, they were present over a wide geographic range (distance), which indicates possible genetic connectivity of the parasite populations. This suggests that the movement of malaria parasites by human reservoirs may connect geographically distinct malaria transmission areas in southern Africa.

While cross-border genetic connectivity between certain countries in the region was already reported in separate studies (7, 10, 27), collating this genetic data with that in the rest of the region would therefore make the regional analysis in the E8 region stronger as a wider geographic scale is covered. Genotype data was collated from 4 (but not widely) and not all 8 countries in the E8 region for the study because of the unavailability of data from the remaining countries on the chosen microsatellite genotyping platform. A major constraint of studies that require direct comparisons from microsatellite markers is that they require the same experimental conditions and genotyping analysis parameters, and data processing settings to be able to be directly compared to each other, as any inconsistencies in data processing settings may potentially distinguish/differentiate between otherwise identical alleles/genotypes (difference of a single allele may be interpreted as a different allele/genotype) (41). With additional microsatellite genetic data from the remaining 4 countries in the E8 region, or other types of data, which may be less prone to the limitations that arise from microsatellite markers, and may have a higher resolution to detect signals of genetic relatedness (such as highly multiplexed amplicon sequencing) (42–44), genetic data of a larger spatial scale can be shared within the region facilitating the identification of origins of imported infections so as to be able to put interventions in place where there is a higher risk of this occurring. A major strength of this current study was the sample size in which a good representation of affected areas was achieved.

The patterns of private allelic richness that suggest an endogenous circulation of parasites in the eliminating countries is an interesting finding which could further hamper elimination efforts in the respective countries. From an elimination perspective, in a low-transmission elimination setting like Eswatini or the KZN Province of South Africa, this shows that although imported infections play a significant role in continued transmission (10, 31), there is also a contribution of locally acquired/generated parasites (parasites of local/internal origin) circulating within the individual countries preventing those countries from “getting to zero” (local) infections. This finding may therefore assist by giving further insights into the behavior of the parasite for example in the MOSASWA malaria cross-border initiative works through harmonized collaborative efforts to achieve zero local transmission in Eswatini, South Africa, and Maputo Province, Mozambique by 2020 and pre-elimination status in southern Mozambique (Maputo and Gaza Provinces) by 2025 (45–47). Alternatively, the observed patterns of private allelic richness may have been due to technical and/or batch effect. The variation seen in the resulting data may have been as a result of technical differences in the way the experiment was performed by the different countries, and/or differences in the batches of samples whereby individual groups of samples were processed differently from each country.

Harmonizing the time and place when appropriate interventions can be deployed is, however, governed by understanding the impact of imported malaria infections on local transmission. In the event of an endogenous circulation of parasites, local control measures such as vector control will be necessary. However, if there is strong genetic connectivity between local and imported infections, then, interventions aimed at decreasing malaria in the source areas of infection or decreasing vulnerability to importation may be required (1, 3).

The results generated in this study are a useful starting point for a future larger study which includes broader representation of countries in the E8 region which will facilitate decision-making for malaria elimination efforts in the region by targeting interventions effectively in both source and sink areas on both a national and regional level thus preventing the continued spread of infections in the region and hopefully achieve elimination. This data can also be used as an early warning system for preventing the undetected spread of for example drug-resistant and imported infections.

## 5. Conclusion

Studying the parasite population genetics in the selected four countries provided a preliminary understanding of parasite genetic connectivity in this area of the Elimination 8 region of Africa, as well as added to the knowledge of understanding local and cross-border malaria transmission dynamics in the region. Results in this study showed strong signals of parasite population structure and genetic connectivity between malaria parasite populations across national borders which calls for strengthening the harmonization of malaria control and elimination efforts between the Elimination 8 countries. This data also proves its potential utility as an additional surveillance tool for malaria surveillance on both a national and regional level as countries work towards malaria elimination. Due to its retrospective nature, this study could not however optimize comparisons between countries due to the different sampling approaches used. It is possible that genetic metrics could be more useful with standardized sampling considering the heterogeneity of malaria transmission. Stratification based on transmission intensity, risk of importation, hotspots, and outbreaks can ensure that genetic analysis is more useful.

## Data Availability

The original contributions presented in the study are included in the article/Supplementary materials, further inquiries can be directed to the corresponding authors.
